# Positive association between moderate altitude and chronic lower respiratory disease mortality in United States counties

**DOI:** 10.1371/journal.pone.0200557

**Published:** 2018-07-11

**Authors:** Jeongeun Hwang, Miso Jang, Namkug Kim, Seunghyun Choi, Yeon-Mok Oh, Joon Beom Seo

**Affiliations:** 1 Asan Institute for Life Sciences, Asan Medical Center, Seoul, Republic of Korea; 2 Department of Family Medicine and Center for Cancer Prevention and Detection, Hospital, National Cancer Center, Goyang, Republic of Korea; 3 Department of Radiology and Research Institute of Radiology, University of Ulsan College of Medicine, Asan Medical Center, Seoul, Republic of Korea; 4 Department of Convergence Medicine, University of Ulsan College of Medicine, Asan Medical Center, Seoul, Republic of Korea; 5 Department of Pulmonary and Critical Care Medicine, University of Ulsan College of Medicine, Asan Medical Center, Seoul, Republic of Korea; University of Calcutta, INDIA

## Abstract

For patients with chronic lower respiratory disease, hypobaric hypoxia at a high altitude is considered a risk factor for mortality. However, the effects of residing at moderately high altitudes remain unclear. We investigated the association between moderate altitude and chronic lower respiratory disease mortality. In particular, we examined the lower 48 United States counties for age-adjusted chronic lower respiratory disease mortality rates, altitude, and socioeconomic factors, including tobacco use, per capita income, population density, sex ratio, unemployment, poverty, and education between 1979 and 1998. The socioeconomic factors were incorporated into the correlation analysis as potential covariates. Considerable positive (R = 0.235; P <0.001) and partial (R = 0.260; P <0.001) correlations were observed between altitude and chronic lower respiratory disease mortality rate. In the subgroup with high COPD prevalence subgroup, even stronger positive (R = 0.346; P <0.001) and partial (R = 0.423, P <0.001) correlations were observed. Multivariate regression analysis of all available socioeconomic factors revealed that additional knowledge on altitude improved the adjusted R^2^ values from 0.128 to 0.186 for all counties and from 0.301 to 0.421 for counties with high COPD prevalence. We concluded that in the lower 48 United States counties, even a moderate altitude may pose considerable risks in patients with chronic lower respiratory disease.

## Introduction

Chronic obstructive pulmonary disease (COPD) and lower respiratory infections are ranked by the World Health Organization as the third and fourth most common causes of death in the world, respectively.[1] In 2020, the number of deaths due to respiratory disease is expected to increase and account for 11.9 of the 68 million total deaths worldwide.[2] However, the causes, potential risk factors, and mechanisms of progression of chronic lower respiratory disease are not completely understood. A number of epidemiologic studies have assessed the prevalence, comorbidities, and risk factors for chronic lower respiratory disease.[3–6] For example, Lewis et al investigated a representative population of the United States (US) and found that low education and low family income were associated with lung cancer and COPD.[4]

Altitude is one of the most fundamental environmental factors that has been reported to have association with diseases, such as cardiovascular disease cancer, COPD, pneumonia, and renal disease.[7–12] The association between altitude and diseases of the respiratory system is pleiotropic. In Mexico, high altitude was shown to have a beneficial association with tuberculosis mortality but had a harmful association with pneumonia and influenza mortality.[11] In Peru, the seemingly protective effect of altitude in tuberculosis was overwhelmed by the population density.[13] Among the studies that investigated the effect of altitude on mortality rates due to chronic lower respiratory disease, a national study on US counties that was conducted by Ezzati et al between 2001 and 2005 found a harmful dose-response association between COPD mortality and altitude,[8] but that study did not include other chronic lower respiratory diseases or pneumonia. Many of the studies concerning altitude and respiratory disease mortality rates were focused on a limited region [14, 15] or on acute reactions to high altitudes, such as acute mountain sickness [16] and high-altitude pulmonary edema.[17–19]

In the present study, we collected the United States mortality statistics for 20 years between 1979 and 1998. We analyzed the association between respiratory disease mortality rates and altitude in the 3,089 counties of lower 48 US, while controlling for potential covariates, such as per capita income, population density, unemployment rate, poverty, education, and sex ratio.

## Materials and methods

### Ethics approval

Ethics approval was not required because this study was performed using a publicly accessible national epidemiology database.

### Mortality statistics

The Centers for Disease Control and Prevention WONDER database was used to extract the US mortality data from 1979 to 1998 by disease, which was classified by the International Classification of Disease (ICD)-9. Diseases of the respiratory system (460–519), pneumonia (480–486), and chronic lower respiratory disease (490–496) were investigated. Chronic lower respiratory diseases included bronchitis, emphysema, asthma, and COPD. Mortality rates were age-adjusted for deaths per 100,000 by standard populations in 2000, and subjects aged <5 years were excluded. County was used as the residential unit, and mortality rates were extracted for every lower 48 US continental county that had available data on altitude. Counties with <20 deaths for a specific mortality code were excluded.

### Altitude

The Shuttle Radar Topography Mission (SRTM) elevation data, which were created by the National Geospatial-Intelligence Agency and the National Aeronautics and Space Administration, were used to determine the average altitudes of the counties, as previously described.[20] SRTM is a global dataset developed in February 2000, with a spatial resolution of approximately 0.1 km. In brief, the average altitude of each county was calculated using zonal statistics in the ArcGIS/ArcInfo 9.3 environment (ESRI, Redlands, CA), which is a geographical information system software that is used to calculate related data on mapping and for querying geographical databases. The data from the SRTM provided the mean altitude calculations for each square kilometer in each county. County boundaries, which were provided by the National Atlas of the United States Geological Survey, were overlaid on the mean spatial data to obtain the mean altitude for each US county. The data inputs for this analysis used a 1:2,000,000 scale for US county vector dataset and a mosaic digital elevation model with approximately 0.5-km spatial resolution, which was derived from the SRTM dataset.

### Socioeconomic factors

Socioeconomic factors, such as per capita income, population density, sex ratio, unemployment, poverty, education, and tobacco use, were considered as potential covariates for mortality. The Area Health Resources Files system was accessed to obtain the unemployment rates for 1990–1998, sex ratio in 2000, percentage of persons in poverty in 2000, and percentage of individuals aged >25 years with <9 years of education in 2000 for every county. Unfortunately, county-specific smoking rates were not available. Instead, the Behavioral Risk Factor Surveillance System was accessed to obtain the current smoking rate per state in 1996. Counties that lacked one or more census data were excluded from the analysis; therefore, 2,678 counties were included in the final analysis.

### COPD prevalence

The data on the COPD prevalence of each county were not available during the study period (1979–1998). Alternatively, we consulted a COPD prevalence study that used the census 2010 data.[21] The exact prevalence rate was not shown, but the COPD prevalence was shown in a five-grade color code on the published figure. From this, 114 counties with the highest (>13%) COPD prevalence were identified and underwent the same analysis as that for all the counties combined.

### Correlation analysis

Pearson’s correlation and partial correlation coefficients between altitude and the mortality rates of the three respiratory disease categories were calculated using normality tests. Partial correlation coefficients were measured while controlling for all of the socioeconomic factors. A multivariate linear regression model was used to assess the contribution of altitude to the explanation power of the models for mortality rates. The adjusted R^2^ values of the multivariate linear regression models were measured while accounting for all the socioeconomic factors without altitude. Next, altitude was added to the multivariate linear regression analysis, and new adjusted R^2^ values were calculated; significance and multicollinearity statistics were measured accordingly. Statistical analyzes were performed using the R statistical software version 3.2.4 (R Foundation for Statistical Computing, Vienna, Austria) and the following R packages: dplyr,[22] ggplot2,[23] maps,[24] and ppcor.[25]

## Results and discussion

**Fig 1:**
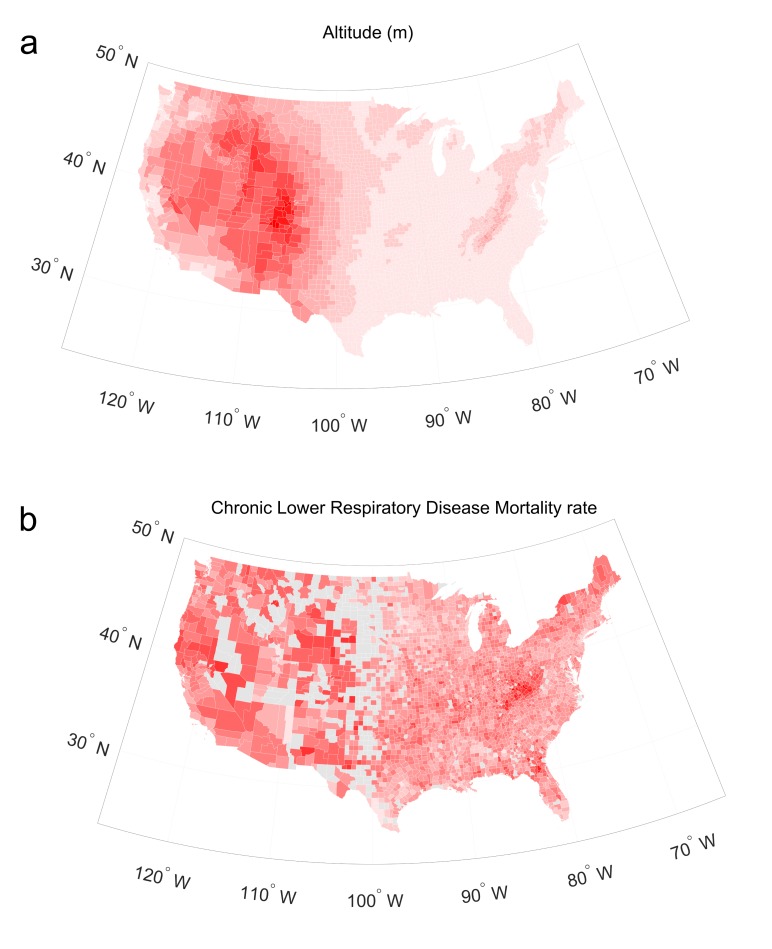
Geographic patterns throughout the continental United States are shown. The patterns for altitude (a) and chronic lower respiratory disease mortality rate (b). Counties with <20 deaths for a specific mortality code and those that lacked one or more census data were excluded from the analysis and are indicated in gray.

Table 1 shows the demographics of all studied counties and those with high COPD prevalence. The geographical patterns of altitude and chronic lower respiratory disease mortality rate are depicted in Fig 1. A considerable positive association was found between the counties’ altitude and chronic lower respiratory disease mortality rate. In Table 2, a simple correlation coefficient of 0.235 and a partial correlation coefficient of 0.260 were observed after controlling for the other covariates, with P values of <0.001. Altitude remained a significant factor in the multivariate regression analysis that incorporated all the socioeconomic factors. The adjusted R^2^ value of the multivariate regression model that included all covariates, except altitude, was 0.128; addition of altitude in the model enhanced the R^2^ value to 0.186. The mortality rates by disease of the respiratory system had a similar correlation with altitude. Pneumonia mortality correlated with altitude, but the intensity of this association and the explanation power were less obvious than those of chronic lower respiratory disease mortality.

**Table 1:** Demographics in all counties and in counties with high COPD prevalence (>13%).

	All counties^a^	Counties with high COPD prevalence^b^
Number of counties	2678	114
Mortality rate: chronic lower respiratory disease^c^	45.1 ± 9.8 (17.8–98.8)	54.9 ± 13.9 (24.5–98.8)
Mortality rate: pneumonia^d^	30.9 ± 7.8 (11.6–75.3)	36.4 ± 8.0 (16.0–56.0)
Mortality rate: disease of the respiratory system^e^	91.2 ± 16.0 (51.6–179.8)	109.6 ± 22.0 (64.5–177.3)
Altitude (m)	375.2 ± 431.5 (0.8–3,041.5)	312.0 ± 304.2 (25.3–2080.5)
Per capita income ($)^f^	18,083 ± 3,973 (7,271–60,297)	14,826 ± 22,26 (10,328–19,637)
Population density^g^	235.1 ± 1535.5 (0.9–53,180.9)	48.0 ± 37.4 (1.1–191.1)
Sex ratio^h^	0.980 ± 0.078 (0.742–1.909)	0.964 ± 0.055 (0.866–1.299)
Poverty rate^i^	16.5 ± 7.8 (2.2–63.1)	24.6 ± 8.8 (10.6–52.1)
Under-education rate^j^	14.5 ± 7.2 (1.1–56.3)	24.7 ± 9.2 (7.4–49.1)
Smoking rate^k^	24.3 ± 2.9 (15.9–31.7)	26.9 ± 3.9 (20.3–31.7)

Data are shown as mean ± standard deviation (range).

^a^Counties that lacked one or more census data were excluded; therefore, 2,678 counties were included in the final analysis.

^b^COPD prevalence of >13% based on the 2010 census [21]

^c^age-adjusted mortality rate per 100,000 in 1979–1998, ICD9 code J40–J47

^d^age-adjusted mortality rate per 100,000 in 1979–1998, ICD9 code J12–J18

^e^age-adjusted mortality rate per 100,000 in 1979–1998, ICD9 code J00–J98

^f^in 1990–1998

^g^in 2000

^h^in 2000

^i^percentage of persons in poverty in 2000

^j^percentage of individuals aged >25 years with <9 years of education in 2000

^k^in 1996

**Table 2:** Correlation coefficients between altitude and mortality rates for diseases of the respiratory system, pneumonia, and chronic lower respiratory disease and adjusted R^2^ values for multivariate linear regression models with or without altitude for all counties.

	Chronic lower respiratory disease	Pneumonia	Disease of the respiratory system
Correlation coefficient	0.235	0.074	0.171
Partial correlation coefficient	0.260	0.148	0.238
Adjusted R^2^ of the regression models that included all covariates^a^ except altitude	0.128	0.090	0.133
Adjusted R^2^ of the regression models that included all covariates and altitude	0.186	0.110	0.182

^a^per capita income, population density, sex ratio, unemployment rate, percentage of persons in poverty, percentage of individuals aged >25 years with <9 years of education. All correlation coefficients and R^2^ values had statistical significance (P <0.001).

**Fig 2:**
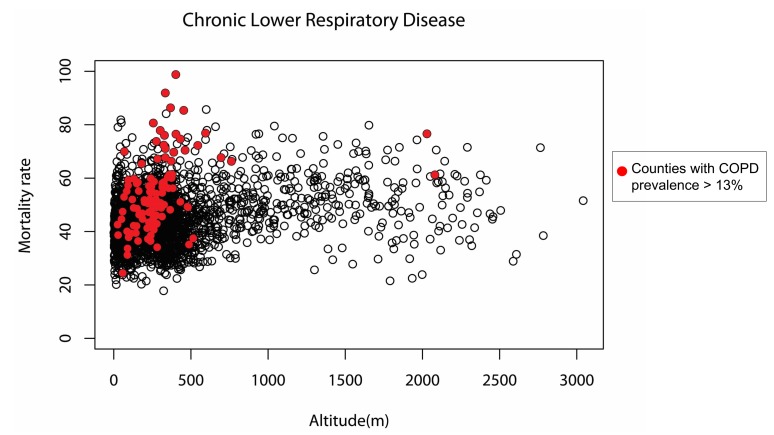
Correlation between altitude and chronic lower respiratory disease mortality rate. A positive correlation was found and was stronger in counties with higher COPD prevalence (>13%; red dots) than in all counties combined (black circles). COPD, chronic obstructive pulmonary disease.

We hypothesized that the correlation between mortality rate and altitude would be more intense in counties where the underlying chronic lower respiratory disease was more prevalent. Considering that the prevalence of COPD reflected that of chronic lower respiratory disease, Fig 2 shows how the association patterns of altitude with mortality differed between COPD-prevalent counties and all counties combined. In the subgroup analysis (Table 3), altitude and chronic lower respiratory disease mortality rate had a simple correlation coefficient of 0.346 and a partial correlation coefficient of 0.423, with p values of <0.001. In the multivariate linear regression analysis, the adjusted R^2^ value was 0.301 for socioeconomic factors alone and improved to 0.421 after adding altitude to the model. The associations between altitude and mortality rate were similar for diseases of the respiratory system and chronic lower respiratory disease but were less significant for pneumonia. The distributions of the socioeconomic factors in COPD-prevalent counties and those in all counites did not significantly differ (S1 Fig).

**Table 3:** Correlation coefficients between altitude and mortality rates for diseases of the respiratory system, pneumonia, and chronic lower respiratory disease and adjusted R^2^ values for multivariate linear regression models with or without altitude for counties with high COPD prevalence (>13%).

	Chronic lower respiratory disease	Pneumonia	Disease of the respiratory system
Correlation coefficient	0.346**	0.214*	0.335**
Partial correlation coefficient	0.423**	0.275*	0.453**
Adjusted R^2^ of the regression models that included all covariates except altitude	0. 301**	0.168**	0.374**
Adjusted R^2^ of the regression models that included all covariates and altitude	0.421**	0.224**	0.497**

* P <0.05

** P <0.001; COPD: chronic obstructive pulmonary disease.

In this study that encompassed almost all US counties during a 20-year period between 1979 and 1998, significant positive associations between altitude and respiratory disease mortality rates were observed. The results were qualitatively consistent with those of previous studies.[8, 11, 14–19, 26, 27] Moreover, this study showed new findings that chronic lower respiratory disease mortality was positively correlated with both extremely high and moderate altitudes. This implied that a moderate altitude, even in ordinary US counties, should not be neglected when dealing with respiratory diseases.

Although the mechanisms by which high altitude affects respiratory disease are not completely understood, several processes were proposed. First, lower oxygen partial pressure [28] and increased solar radiation at high altitudes are generally detrimental [8] for COPD and other respiratory diseases. In addition, climatic factors, such as low temperature, low humidity, and high wind velocity, which are found at high altitudes, worsened asthma by increasing bronchial hyperresponsiveness and inflammation.[28, 29] Latitude is another geographical factor that is closely related with temperature; however, it did not have significant associations with any of the three respiratory disease categories in our study. Considering that the counties investigated in this study had latitudes that ranged from 25 to 45 degrees from the subtropical to the subpolar regions, the association of altitude with mortality could not be discounted as a mere result of temperature variations. Low absolute and partial pressures of oxygen can inhibit lung fluid absorption and lead to acute pulmonary edema.[17–19] Residents of places with relatively high altitudes might undergo chronic, progressive inhibition of lung fluid absorption and become more susceptible to respiratory infections. A simulation study showed that patients with chronic airway obstruction exhibited pulmonary dysfunction and worse hypoxemia in atmospheric conditions that mimicked altitudes of ≥1,524 meters.[30] Furthermore, chronic hypoxemia induced leukocyte-mediated tissue damage.[31] Therefore, the pathophysiologic response of lung tissue to hypoxia, might be mediated by hypoxia inducible factor-1 [32–35] or nitric oxide [36, 37], could be detrimental to a diseased lung.

In the present observational study, determination of a causal association between mortality rate and altitude and of the exact aspect of low altitude that was harmful for respiratory diseases was difficult. This limitation raises the need for further studies.

### COPD prevalence

The stronger correlation between altitude and mortality rate in counties with high COPD prevalence (Fig 2 and Table 2) can lead us to speculate that a diseased lung is susceptible to further destruction at moderate altitudes. If the prevalence data for chronic lower respiratory disease in each county were available at the time of this study, this speculation could have been more thoroughly investigated.

### Air quality

Air pollution is a significant risk factor for respiratory disease [38–40], and the public tends to believe that high-altitude ranges have cleaner air, compared with low-altitude areas. We investigated the covariates that represented the extent of air pollution, daily fine particulate matter, and days with an eight-hour average ozone over National Ambient Air Quality Standards, but we could not determine significant associations between air pollution and mortality rates (data not shown). There were reports that the associations among altitude, air quality, and respiratory disease were equivocal or not simply proportional [41–43]; however, we did not observe these in our current study. Further studies are warranted to resolve the cause-and-effect associations among these factors.

### Limitations

Other potential confounding factors, such as county-wise wind chill temperature, background radiation, ethnicity, medical care system, utilization of preventive measures, and industrial background of the counties, were not included in the analysis. Considerable variations in mortality rate, altitude, prevalence, and socioeconomic status within a county were also not accounted for in the present study. Another limitation of the present study was a study period mismatch among mortality statistics (1979–1998), unemployment rate (1990–1998), sex ratio (2000), poverty (2000), education (2000), smoking rate (1996), and COPD prevalence (2010). Although this mismatch was inevitable, it may add some complexity in the interpretation of the results, because there could have been considerable changes in between the time gaps. To clarify the mechanism underlying the association between altitude and chronic lower respiratory disease mortality rate, further studies are required. Nevertheless, this investigation across the entire US over 20 years should partially compensate for the limitations of these confounding factors.

## Conclusions

In the US, there was a positive association between altitude and mortality rate from respiratory diseases, especially for chronic lower respiratory disease. In counties with high COPD prevalence, the harmful association was even stronger than that in the other counties. There is a need for further studies on the underlying mechanism of this association.

## Supporting information

S1 Fig Histogram of the socioeconomic factors in all counties (dark gray) and in COPD-prevalent counties (light gray). The distribution patterns did not differ significantly. COPD, chronic obstructive pulmonary disease.
(JPG)

S1 Table Correlation coefficients between all variables and the mortality rates for chronic lower respiratory disease, according to county and state. (DOC)
