# Supplementary material for: Positive association between moderate altitude and chronic lower respiratory disease mortality in United States counties
Source: PLoS One. 2018 Jul 11;13(7):e0200557. doi: 10.1371/journal.pone.0200557 (PMC6040762; doi:10.1371/journal.pone.0200557)
Supplement: S1 Table — (DOC) [file pone.0200557.s002.doc]

**Positive association between moderate altitude and chronic lower respiratory disease mortality in United States counties**

Jeongeun Hwang, Miso Jang, Namkug Kim, Seunghyun Choi, Yeon-Mok Oh, Joon Beom Seo

**S1 Table. Correlation coefficients between all variables and the mortality rates for chronic lower respiratory disease, according to county and state**

| Chronic lower respiratory disease mortality rate | States | | Counties | |
| --- | --- | --- | --- | --- |
|  | R | P | R | P |
| Altitude | 0.541 | <0.001 | 0.235 | <0.001 |
| Latitude | -0.037 | 0.803 | -0.051 | 0.006 |
| Sex ratio | 0.598 | <0.001 | 0.148 | <0.001 |
| Population density | -0.424 | 0.002 | -0.114 | <0.001 |
| Smoking prevalence | 0.375 | 0.008 | - | - |
| Per Capita Income | -0.342 | 0.016 | -0.215 | <0.001 |
| Unemployment rate | 0.194 | 0.182 | 0.185 | <0.001 |
| Poverty | 0.102 | 0.486 | 0.126 | <0.001 |
| Under-education | -0.048 | 0.743 | 0.049 | 0.007 |
| Fine particulate matter | -0.003 | 0.985 | 0.051 | 0.005 |
| Ozone | -0.274 | 0.057 | 0.005 | 0.790 |

States: based on 48 states and the District of Columbia (excludes Alaska and Hawaii); Counties: Based on 2,678 to 2,965 counties (excludes Alaska and Hawaii; counties with <20 deaths for a specific mortality code; counties that lacked census data); R: Pearson’s correlation coefficient; P: p-value; Sex ratio: male/female in 2000; Population density: population per square mile in 1992; Smoking prevalence: current smoking rate in 1996; Per Capita Income: in 1990–1998; Unemployment rate: in 1990–1998 (persons/persons); Poverty: per persons below poverty level in 1989; Under-education: per persons older than 25 years with less than 9 years education in 1990; fine particulate matter: Daily fine particulate matter average in 2008–2011; Ozone: days with eight-hour average ozone over NAAQS in 2006–2011;
